# Thermodynamic Analysis of Size-Dependent Surface Energy in Pd Nanoparticles for Enhanced Alkaline Ethanol Electro-Oxidation

**DOI:** 10.3390/nano14231966

**Published:** 2024-12-07

**Authors:** A. Santoveña-Uribe, J. Maya-Cornejo, M. Estevez, I. Santamaria-Holek

**Affiliations:** 1 Unidad Multidisciplinaria de Docencia e Investigación-Juriquilla, Facultad de Ciencias, Universidad Nacional Autónoma de México (UNAM), Juriquilla, Querétaro 76230, Mexicoisholek@ciencias.unam.mx (I.S.-H.); 2 Centro de Física Aplicada y Tecnología Avanzada, Universidad Nacional Autónoma de México (UNAM), Juriquilla, Querétaro 76230, Mexico

**Keywords:** palladium, ethanol oxidation, nanoparticles, surface energy, electrocatalysis, fuel cells

## Abstract

This work investigates the relationship between the mean diameter of palladium (Pd) nanoparticles and their surface energy, specifically in the context of alkaline ethanol electro-oxidation for fuel cell applications. Employing a recent generalization of the classical Laviron equation, we derive crucial parameters such as surface energy (*σ*), adsorption–desorption equilibrium constant (*K_eq_*), and electron transfer coefficient (*α*) from linear voltammograms obtained from Pd-based nanoparticles supported on Vulcan carbon. Synthesized using two distinct methods, these nanocatalysts exhibit mean diameters ranging from 10 to 41 nm. Our results indicate that the surface energy of the Pd/C nanocatalysts spans *σ* ~ 0.5–2.5 J/m^2^, showing a linear correlation with particle size while remaining independent of ethanol bulk concentration. The adsorption–desorption equilibrium constant varies with nanoparticle size (~0.1–6 × 10^−6^ mol^−1^) but is unaffected by ethanol concentration. Significantly, we identify an optimal mean diameter of approximately 28 nm for enhanced electrocatalytic activity, revealing critical size-dependent effects on catalytic efficiency. This research contributes to the ongoing development of cost-effective and durable fuel cell components by optimizing nanoparticle characteristics, thus advancing the performance of Pd-based catalysts in practical applications. Our findings are essential for the continued evolution of nanomaterials in fuel cell technologies, particularly in improving efficiency and reducing reliance on critical raw materials.

## 1. Introduction

The transition to sustainable energy systems is increasingly reliant on the development of efficient fuel cell technologies, particularly in sectors where electrification is not feasible. Alkaline fuel cells, utilizing hydrogen or alcohols like ethanol as fuel, have garnered significant attention due to their high efficiency and potential for low-cost production. Among the various materials explored for fuel cell catalysts, palladium (Pd) nanoparticles have emerged as promising candidates due to their superior electrocatalytic properties, especially in the electro-oxidation of ethanol [1,2,3,4].

In this sense, the oxidizing alcohols in polymer electrolyte membrane fuel cells (PEMFCs) and, keeping in mind the oxygen reduction reaction, the physicochemical modifications of electrocatalytic materials have been focused on generating different kinds of morphologies [5,6,7,8,9,10], surface defects [11,12,13,14], and geometric structures of active sites [15,16], and reducing the size of the particles [17,18,19,20,21]. Although studies extended to other electrocatalytic reactions have also been performed [22,23], the efforts to reduce the particle size have been based on the premise that, when the particle size decreases, the relative surface area increases [24], improving in this way the electrocatalytic activity [25,26,27,28,29]. 

However, very small particle sizes may induce steric effects and entropic restrictions on the electrocatalytic activity. For instance, when a sufficiently long alcohol molecule (like methanol or ethanol) reaches the particle surface, hindrance between alcohol molecules during the adsorption process may appear, so that the oxidation process becomes inhibited, and the catalytic efficiency of the nanoparticle diminishes. The importance of this work rests on defying the widely extended popular belief that “smaller is better”, demonstrating an optimal particle size from a theoretical approach supported by an experimental study.

To clarify the validity of this premise, we compare the electro-oxidation of ethanol by Pd/C-based electrocatalysts of diameters ranging from 10 to 41 nm in alkaline media [30,31]. This assumption was based on evidence from a prior study [29], which used a generalized Laviron equation derived in that same study [29,32] to analyze the electrochemical response of Pd/C- and Cu@Pd/C nanocatalysts.The determination of the ethanol adsorption/desorption equilibrium constant over Pd/C and Cu@Pd/C nanocatalysts was performed assuming a fixed reference value of the surface energy, reported in the literature, leading to an irregular behavior of the ethanol equilibrium constant as a function of the bulk ethanol concentration. However, the almost linear dependence of the number of adsorbed particles on the nanocatalyst surfaces in terms of the ethanol bulk concentration and the irregular behavior of the adsorption/desorption equilibrium constant later suggested that the surface energy of the nanocatalysts may depend upon their size.

Due to the earlier discussion, the actual study is also predicated on the hypothesis that a better distribution of nanoparticles with optimal sizes over the carbon support proves to be essential for their practical performance to prevent steric effects and entropic restrictions on the electrocatalytic activity. This approach shares certain similarities with the use of Metal Oxide Frameworks (MOFs), which are capable of efficiently absorbing and transporting molecules. The huge number of cavities in these arranged materials behaves like a very small sponge, giving an exceptional support surface area along which nanoparticles with optimized sizes could be distributed [33,34]. 

In a concrete way, this study aims to elucidate the relationship between nanoparticle size and surface energy in Pd-based catalysts specifically for alkaline ethanol electro-oxidation. By employing a generalized form of the classical Laviron equation [29,32], we seek to derive critical parameters such as surface energy (*σ*), adsorption–desorption equilibrium constant (*K_eq_*), and electron transfer coefficient (*α*) from linear voltammetry data. Through this research, we propose that there exists an optimal mean diameter that enhances catalytic efficiency, thus contributing valuable insights for the design of effective Pd-based electrocatalysts for fuel cell applications. In this perspective, we use two different Pd/C synthesis methods to avoid the shape factor that could slightly modify the Ethanol Oxidation Reaction [35], which therefore allows us to assume an approximated sphericity of the nanoparticles. By focusing primarily on electrochemical measurements, we seek to describe the relationships between particle size, surface energy, and catalytic performance, which can be described by our proposed modified version of the classical Laviron equation.

## 2. Theoretical Framework 

In a previous work, we derived a generalized version of the Laviron equation [29,32] that provides an explicit expression of the number of alcohol molecules adsorbed over the surface of the nanoparticle in terms of the alcohol bulk concentration and of the surface energy of the nanocatalysts. The result incorporates the well-known fact that thermodynamic properties, like the fusion temperature or the chemical potential, become size-dependent for nano-scaled structures. In this form, the modified Laviron equation becomes:

(1) $$i(E)=\frac{F^{2}}{RT}n(1-\alpha )\nu \Gamma_{\sigma }e^{\frac{(1-\alpha )F}{RT}(E-E_{p})}e^{-e^{\frac{(1-\alpha )F}{RT}(E-E_{p})}}$$

where the dependence on the surface energy (*σ*) of the nanocatalyst is contained in the modified number of adsorbed molecules, which is given by the formula:

(2) $$\Gamma_{\sigma }=\frac{K_{eq}e^{\frac{4\sigma \bar{V_{p}}}{DRT}}C_{M}^{0}C_{A}}{C_{M}^{0}+C_{A}\left(1+K_{eq}e^{\frac{4\sigma \bar{V_{p}}}{DRT}}\right)}$$

Equation (2) modifies the Laviron expression by considering that catalyst nanoparticles have a finite diameter *D* and, therefore, their chemical affinities become modified by the associated surface energy *σ*. Therefore, evaluating Equation (1) in the peak of the oxidation current, that is, for *E* = *E_p_*, we obtain:

(3) $$i_{p}=\frac{F^{2}}{RT}n(1-\alpha )\nu \frac{K_{eq}e^{\frac{4\sigma \bar{V_{p}}}{DRT}}C_{M}^{0}C_{A}}{C_{M}^{0}+C_{A}\left(1+K_{eq}e^{\frac{4\sigma \bar{V_{p}}}{DRT}}\right)}$$

Equation (1) describes the oxidation current obtained in terms of the applied voltage. This current depends now on the key properties determining the adsorption process, such as the equilibrium constant (*K_eq_* = *Γ_σ_*/*C_A_*), where *Γ_σ_* is the number of molecules adsorbed on the nanoparticle surface in mol, the surface energy (*σ*) in J/m^2^, the initial concentration of active sites ($C_{M}^{0}$) in mol, the bulk concentration of alcohol (*C_A_*) in mol, and the parameter *D* in nm, which is an estimate of the particle size assuming it is a sphere. This sphericity assumption seems reasonable when the tested particles are larger than a few nanometers, as we are interested in. In a more general case, the term accounting for the surface energy in Equation (1) can be written as the sum of the surface energies of different crystal facets divided by a characteristic length. Thus, the surface energy and the particle size represent the mean value of the total surface energy of the nanoparticle.

Now, it is worth noticing that the total number of active sites on the surface of the nanoparticles $C_{M}^{0}$ (defined by the Pd ECSA, the total metal weight deposited on the working electrode, and the particle area) is larger than the bulk alcohol concentration adsorbed on each site *C_A_* (obtained from the total concentration ${C_{T}={(C}_{A}+C}_{M}^{0}$). Since the equilibrium constant is expected to be a small number [29], we can assume that the total number of active sites is much larger than the number of occupied sites and therefore:

(4) $$C_{M}^{0}\gg C_{A}\left(1+K_{eq}e^{\frac{4\sigma \bar{V_{p}}}{DRT}}\right)$$

The values of the adsorption–reaction constant (*K*_*eq*_) and the surface energy (*σ*) of the studied nanoparticles can be determined from Equation (3). After using the assumption presented in Equation (4), the peak current in Equation (3) takes the simple form:

(5) $$i_{p}=i_{0}e^{\frac{4\sigma \bar{V_{p}}}{DRT}}$$

where *i*_0_ is a characteristic current that includes constants and alcohol-independent concentration parameters:

(6) $$i_{0}=\frac{F^{2}}{RT}K_{eq}n(1-\alpha )\nu e^{-1}C_{A}$$

Equations (5) and (6) predict that the peak current depends linearly on the bulk alcohol concentration. Additionally, the surface energy also depends linearly on the particle size and logarithmically on the peak current. This equation allows us to determine the surface energy and the equilibrium constant based on the values of the sweep velocity, half-peak current, half-peak potential, peak current, peak potential, concentration of alcohol, and temperature used in experiments. In the following sections, we will return to this subject and describe the algorithm used for this purpose.

**Table 1:** Symbology used and descriptions.

Symbol	Units	Description
*i*	A	Experimental current
*i_p_*	A	Peak current
*i*_0_	A	Characteristic current
*E*	V	Experimental potential
*E_p_*	V	Peak potential
*F*	C/mol	Faraday constant (96,500 C/mol)
*R*	J/molK	Ideal gas constant (8.314472 J/molK)
*T*	K	Temperature (298 K)
*n*	-	Number of transferred electrons (12 e^−^)
*α*	-	Electron transfer coefficient (0.7)
*ν*	V/s	Scan rate (20 mV/s)
*Γ*_0_	mol	Adsorbed molecules
*D*	m	Nanoparticle diameter
$\bar{V_{p}}$	m^3^/mol	Molar volume (8.86 × 10^−6^ m^3^/mol)
*σ*	J/m^2^	Surface energy
$C_{M}^{0}$	mol	Initial concentration of metallic active sites
*C_A_*	mol	Alcohol concentration
*C_T_*	mol	Total concentration

## 3. Materials and Methods

### Synthesis of Pd/C Electrocatalysts

To evaluate the validity of Equation (5), we followed two different methods for Pd-nanoparticle synthesis: polyol synthesis [36,37] and acid ascorbic reduction [29]. In both cases, the following reagents were employed: Polyvinylpyrrolidone (PVP, mol wt. 40,000, Sigma-Aldrich, Darmstadt, Germany), ethylene glycol (EG, 99.8%, Sigma-Aldrich, Darmstadt, Germany), ascorbic acid (AA, 99.0%, Sigma-Aldrich, Darmstadt, Germany), potassium tetrachloropalladate (II) K_2_PdCl_4_, 99%, Sigma-Aldrich, Darmstadt, Germany), Vulcan carbon XC-72R (Cabot, Boston, MA, USA), ethanol (96%, industrial grade) for the dispersion of the carbon support, ethanol (99.7%, Sigma-Aldrich, Darmstadt, Germany) as fuel, Nafion® (5 wt.% in isopropanol, Sigma-Aldrich, Darmstadt, Germany), potassium chloride (99.7%, Sigma-Aldrich, Darmstadt, Germany) to saturate the reference electrode dissolution, and potassium hydroxide (KOH 85%, Sigma-Aldrich, Darmstadt, Germany) were used as received.

To obtain different nanoparticle sizes, the Pd/C electrocatalysts were synthesized employing two different chemical reduction methods. The first method is a modified polyol synthesis: in a 180 °C silicon oil bath and with constant stirring, 4 mL of EG was heated in a 3-necked round-bottom flask connected to a condenser. To obtain different nanoparticle sizes, 1 mL, 2 mL, 3 mL, and 4 mL of K_2_PdCl_4_ (50 mM) dissolved in EG and the same volumes of PVP (100 mM) in EG as a surfactant (Table 1) were added to the reaction at a 25 mL/min rate. Once the solution was finished, the reduction reaction was maintained at the same temperature and stirring conditions for one hour and later quenched to room temperature in a cold recipient. The obtained solutions were washed several times with isopropyl alcohol. For the powder elaboration, 0.4 mg of the cleaned nanoparticle solution and 1.6 mg of Vulcan carbon were placed together in a beaker with some isopropyl alcohol drops in an ultrasound bath, and we let them dry at room temperature.

Regarding the second Pd synthesis method employed, a PVP solution as a surfactant and 10 mL of EG as reaction medium were placed in a round-bottom flask connected to a condenser at a constant stirring rate. The temperature was raised to around 80 °C, and when the PVP was completely dissolved in the EG, 0.06 g of K_2_PdCl_4_ was added. Once the temperature reached 80 °C, 0.127 g of ascorbic acid was added. The palladium reduction reaction was maintained under constant stirring at 80 °C for one hour and a half. Later, 60 mg of Vulcan carbon was dissolved in 50 mL of ethanol and stirred for 30 min to disperse all the Vulcan carbon. After this step, when the reduction of the palladium was carried out, the synthesis was added, drop by drop, to the dispersed Vulcan carbon. The resulting powder was washed several times with distilled water and dried at room temperature overnight. The modification of Pd particle sizes was carried out by modifying the amount of PVP (surfactant) during each synthesis, as is presented in Table 2.

## 4. Material Characterization

### 4.1. Scanning Electron Microscopy (SEM) Characterization

The morphological and structural characterization of the samples was carried out using scanning electron microscopy (SEM) and bright-field scanning transmission electron microscopy (BF-STEM). SEM images were obtained using a SU8230 cold field emission (CFE) SEM/STEM microscope Hitachi, Tokyo, Japan at 30 keV accelerating voltage at 8 mm with the Z contrast STEM and, in some samples, secondary electrons in the upper detector (SE-U). The data acquired to elaborate the mean size histograms of the particles were obtained through contrast differences in ImageJ 1.8.0 software from 250 to 500 manual measures.

### 4.2. Electrochemical Characterization

The electrochemical profiles and the electrocatalytic activity for the ethanol electro-oxidation reaction (EOR) in alkaline media were carried out with linear sweep voltammetry and cyclic voltammetry as electrochemical techniques using a BioLogic Seyssinet-Pariset, France VSP Potentiostat/Galvanostat. The electrochemical experiments were obtained using a three-electrode electrochemical cell configuration in which a glassy carbon (Basi^®^ West Lafayette, IN, USA, 0.0769 cm^2^) was used as the working electrode, a calomel electrode saturated with KCl (SCE) was used as the reference electrode, and a graphite rod was used as the counter electrode. The catalytic inks to test the materials in alkaline media and ethanol were prepared by mixing 1 mg of catalytic powder, 73 μL of isopropanol as a dispersant, and 7 μL of Nafion^®^ Sigma-Aldrich, Darmstadt, Germany as a binder. After stirring, ink was deposited dropwise in the working electrodes using 10 μL to cover the entire glassy carbon electrode zone. The electrochemical profiles were obtained using 0.6 M of KOH dissolution as an electrolyte in the absence of ethanol, while the EOR tests were carried out using 1 M of KOH dissolution and varying the ethanol concentration (0.2 M, 0.5 M, 0.8 M, 1 M, 1.2 M, and 1.5 M). Cyclic voltammograms were obtained in a potential range from −0.6 to 0.8 V vs. NHE (normal hydrogen electrode = SCE + 0.241 V). The scan rate was 20 mVs^−1^ for the electrocatalytic evaluation. The electrolytic solutions were bubbled with nitrogen gas for 10 min (Infra Estado de México, México, 99.999%) before each experiment.

## 5. Results and Discussion

**Figure 1:**
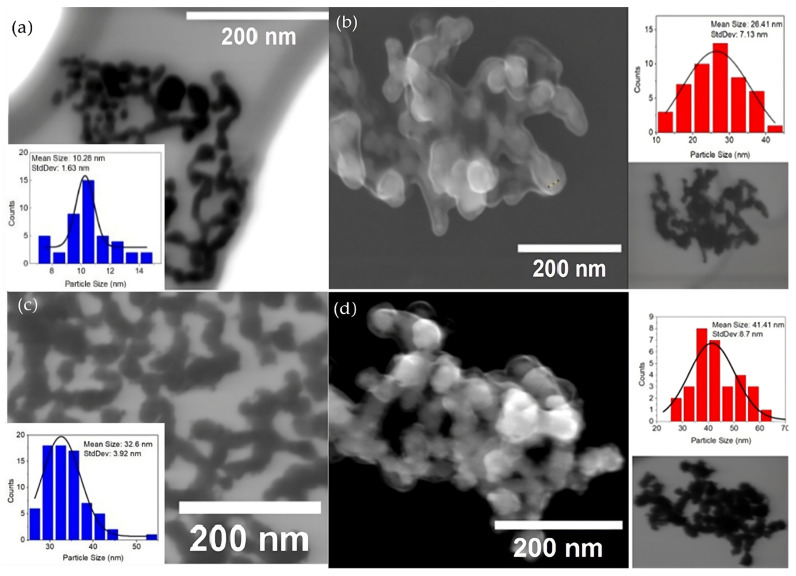
Micrographs of Pd catalysts obtained from the polyol method, without the addition of Vulcan carbon: (**a**) 10 ± 1 nm, BF-STEM; (**b**) 26 ± 7 nm, SE(U) SEM and BF-STEM; (**c**) 32 nm ± 4 nm, BF-STEM; and (**d**) 41 nm ± 9 nm, SE(U) SEM and BF-STEM.

**Figure 2:**
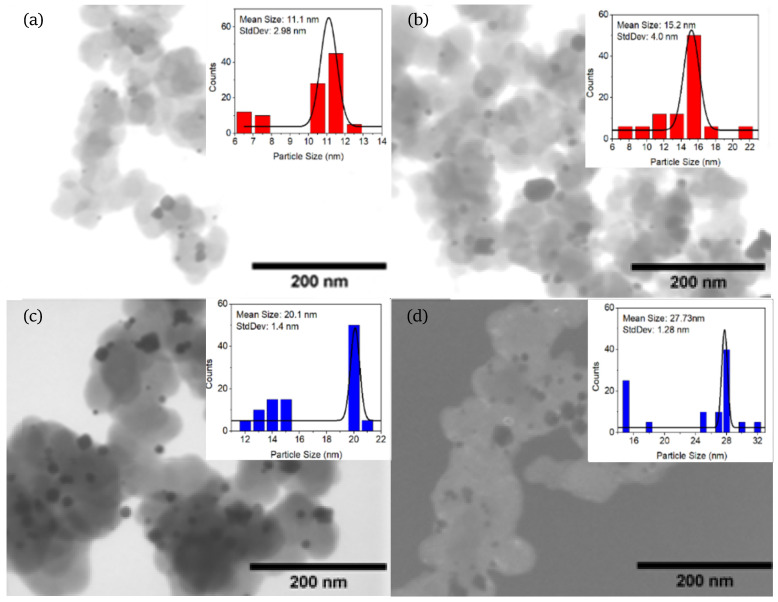
Micrographs of Pd/C electrocatalysts for different particle sizes with ascorbic acid: (**a**) 11 ± 3 nm, (**b**) 15 ± 4 nm, (**c**) 20 ± 1.5 nm, and (**d**) 28 ± 1.5 nm.

**Figure 3:**
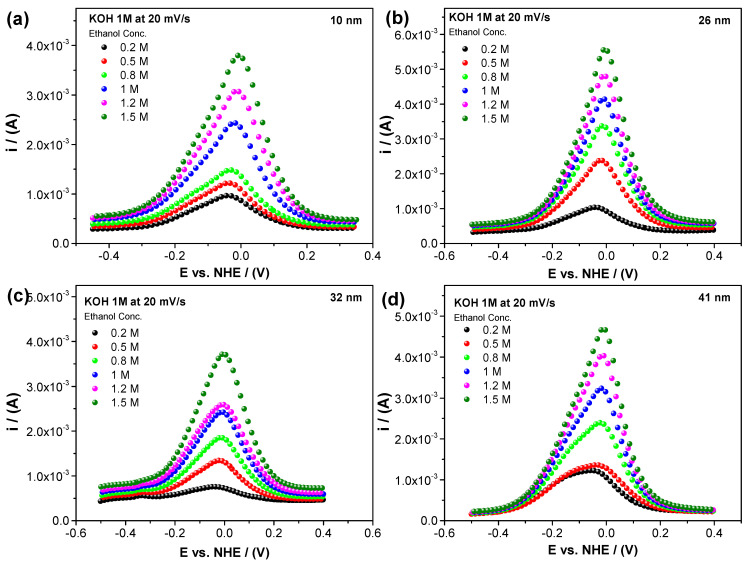
Linear sweep voltammetry for the electro-oxidation of ethanol in an alkaline medium (KOH 1 M) at a scan rate of 20 mV/s using electrocatalysts with a (**a**) 10, (**b**) 26, (**c**) 32, and (**d**) 41 nm particle size (polyol).

First, to determine the size of samples from different synthesis methods, scanning electron microscopy (SEM) and scanning transmission electron microscopy (STEM) studies were performed. Figure 1 and Figure 2 show BF-STEM and SE(U) SEM micrographs for the Pd with no Vulcan carbon catalysts obtained from the polyol method. The Pd/C nanoparticles are embedded in a PVP external layer and still can be better measured on SEM images. The histograms show different particle sizes of 10, 26, 32, and 41 nm, respectively. The BF-STEM micrographs for the Pd/C electrocatalysts obtained from the ascorbic acid method with different particle sizes are shown in Figure 3. To obtain different particle sizes, the amount of PVP was modified: (a) 11, (b) 15, (c) 20, and (d) 28 nm. The histogram presents the distribution of the different particle sizes over the Vulcan carbon support.

**Figure 4:**
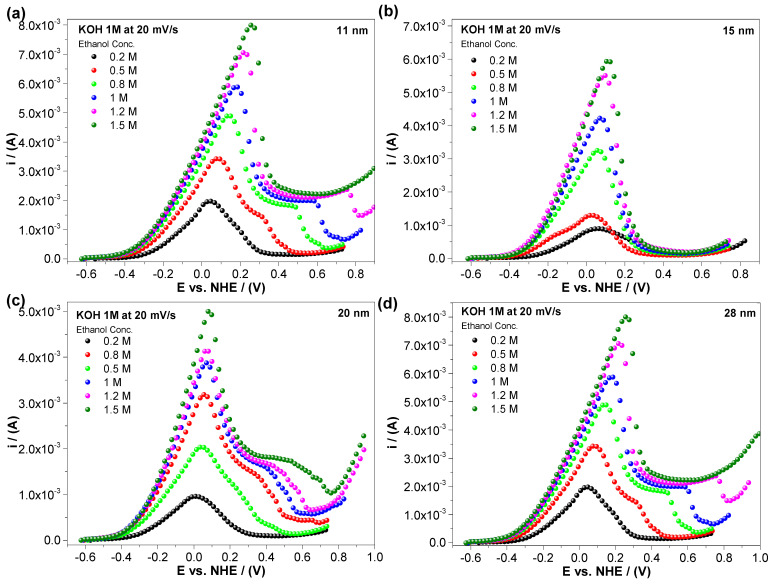
Linear sweep voltammetry for the electro-oxidation of ethanol in an alkaline medium (KOH 1 M) at a scan rate of 20 mV/s using electrocatalysts with an (**a**) 11, (**b**) 15, (**c**) 20, and (**d**) 28 nm particle size (ascorbic acid).

The number of adsorbed alcohol molecules affects the electrocatalytic activity for both the polyol (Figure 3) and ascorbic acid (Figure 4) Pd/C methods of synthesis. The electrochemical response of the ethanol electro-oxidation shows a peak current increment as the alcohol concentration increases (0.2 M, 0.5 M, 0.8 M, 1 M, 1.2 M, and 1.5 M). It can be noticed that the current values are very similar and not normalized, consistent with the modified Laviron equation. As expected at some ethanol concentrations, the alcohol dissolution reaches saturation near the nanoparticle surface, inhibiting the adsorption process. We reached this point at a 1.5 M ethanol concentration, where higher concentration plots exhibited a lower or a very similar peak current, breaking the linear behavior. Higher ethanol concentrations reak the linear trend and were not considered in the present study.

It is important to note that, in the cyclic voltammetry results obtained, there is no significant difference in the peak current regardless of the synthesis method used, demonstrating that the particle size determines the number of active spaces available and, therefore, the surface energy. 

For the theoretical analysis and interpretation of the voltammograms, we performed several fits of the data using Equation (1). For this, we used iteration sequences of fits to the results to feed the modified Laviron equation and obtain a confident fit of the experimental curves and parameter values. The fitting algorithm consists of using the experimental values from voltammograms and Equation (1) in the approximation, where the peak current is given by Equation (3). The first iteration sequence calculates the electron coefficient transfer (*α*) and the number of adsorbed molecules (*Γ_σ_*). A second step involves *α* and *Γ_σ_* recently calculated in a new iteration sequence of Equation (1), evaluated in the same peak current to obtain the adsorption/desorption equilibrium constant (*K*_*eq*_) and the surface energy (*σ*). The experimental values and calculated results are reported, respectively, in Supplementary Information Tables S1 and S2.

**Figure 5:**
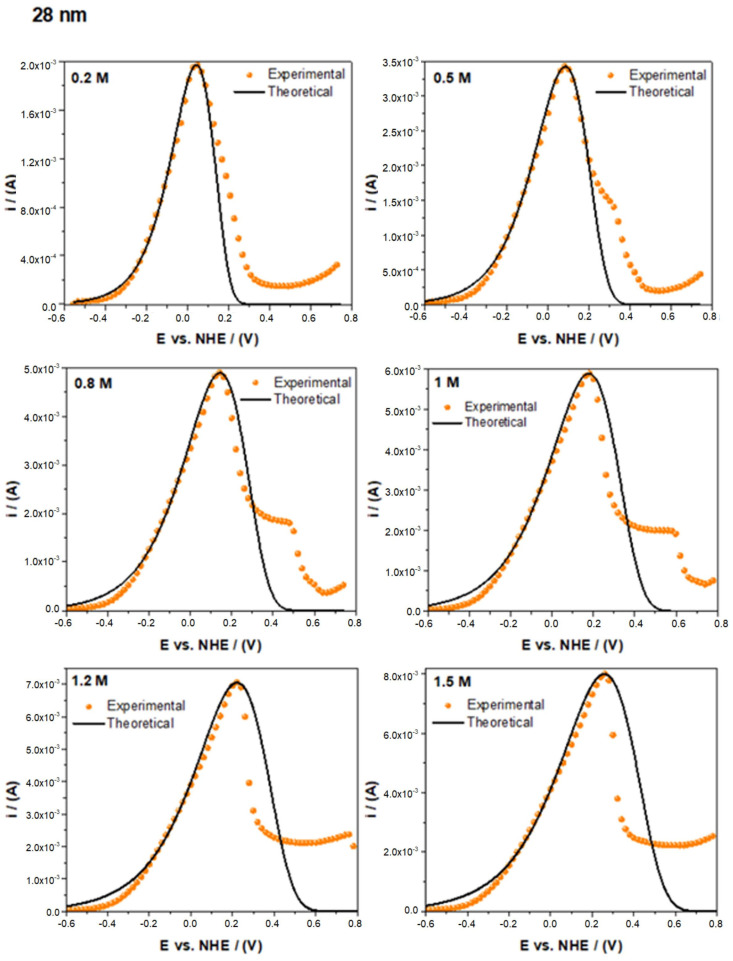
The best experimental data and theoretically fitted values for electro-oxidizing ethanol in an alkaline medium (KOH 1 M) at a scan rate of 20 mV/s were found for the 28 nm sample.

In Figure 5, we show the voltammograms with the theoretical and experimental values of the 28 nm nanoparticle mean size. For all ethanol concentrations in the steady-state oxidation zone (from the equilibrium potential to the peak potential), it was seen that the theoretical curve (black line) fits the experimental results (dots). In this region, the EOR process is governed by two primary factors: The quantity of ethanol *C_A_*, which invariably results in the formation of by-products, and the number of available active sites $C_{M}^{0}$, which, according to the reaction mechanism described in the literature, could be described in cyclic voltammetry profiles as the location where the Pd adsorbed the hydroxyl ions Pd-OH_ads_ in ethanol electro-oxidation CV occupied by a single alcohol molecule Pd-(CH_3_CH_2_OH)ads and later bonded with another by-product like ethoxy or (CH_3_CO)ads [4], preventing other fresh ethanol molecules from adsorbing on. 

This is the part of the oxidation peak that is consistent with the Laviron approach. Above the peak potential region, the theoretical curves do not fit at all the experimental values due to the strong interactions between the palladium oxide (Pd-O) formed in the active sites, the by-products from the electro-oxidation of ethanol as the ethoxy [4], and carboxyl’s capability for the CO oxidation [8]. However, the results show a reasonably good theoretical fitting of the experimental data. All the electrochemical and physicochemical parameters that we obtained from the modified Laviron provide a useful understanding of the electrochemical behavior of ethanol electro-oxidation, as will be clear in the following paragraphs. These lead to the modification of electrocatalytic materials to reach better properties and, finally, increase the electrocatalytic activity. The complete data we obtained are shown in Supplementary Information Table S2.

**Figure 6:**
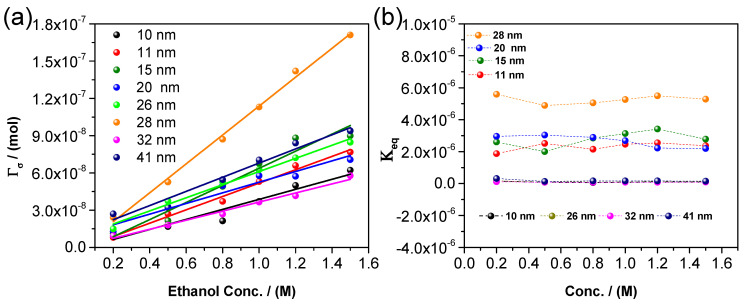
(**a**) Number of molecules adsorbed vs. alcohol concentration and (**b**) adsorption–reaction constant vs. alcohol concentration in alkaline media.

The results obtained for the number of adsorbed molecules (*Γ_σ_*) and the adsorption–reaction constant (*K*_*eq*_) versus ethanol concentration are shown in Figure 6. A general behavior is observed for all the different particle sizes where the number of adsorbed molecules reaches higher values as the concentration of ethanol increases.

As expected, the number of molecules adsorbed on the active sites could be higher by increasing the concentration of alcohol molecules surrounding the nanoparticles (see Figure 6a and data from Supplementary Information Table S2). The linear behavior observed in this figure is predicted by the previous theoretical analysis since Equations (2), (4), and (5) imply a linear relation with the alcohol concentration $\Gamma_{\sigma }≅C_{A}$. Figure 6a shows the number of molecules adsorbed on the active sites of the nanocatalyst (symbols) obtained by fitting the voltammograms of Figure 5. Figure 6b shows that the equilibrium constant (*K*_*eq*_) weakly depends on the characteristic particle diameter due to the polydispersity of each sample and is independent of the alcohol concentration.

For smaller particles, the adsorbed ethanol molecules on the active sites inhibit the adsorption of other molecules. This behavior is observed in particles smaller than 28 nm, suggesting that the active sites suffer from a form of blocking that could be attributed to other ethanol molecules, Pd-oxidation sites, or other by-products from the ethanol electro-oxidation. For nanoparticles larger than 28 nm, the number of active sites on their surface also increases. Nonetheless, as their volume increases, the number of nanoparticles decreases, indicating the presence of a critical size. These results indicate that the number of adsorbed molecules is higher for particle sizes of 28 nm and suggest, in turn, that the system presents a higher contribution of the adsorption–reaction, given that the adsorption–reaction equilibrium constant is the ratio of the adsorbed and desorbed of the ethanol molecules on active sites [9]. This shift promotes the adsorption of molecules when the particle size increases at a certain level, showing a clear particle size effect, allowing both the number of alcohol molecules adsorbed and the increasing peak current to have a direct effect on the electrocatalytic activity.

**Figure 7:**
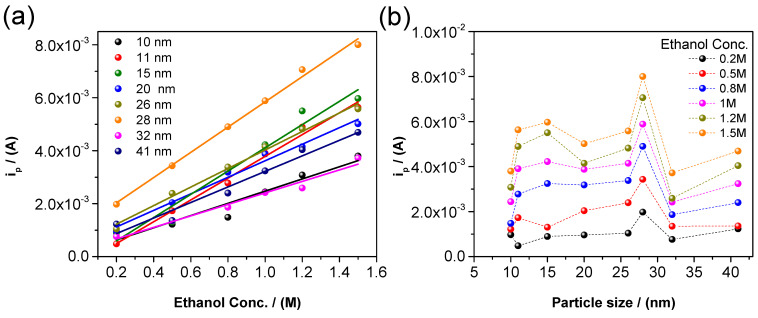
(**a**) Peak current vs. different ethanol concentrations and (**b**) peak current vs. different particle sizes in alkaline media.

Our fitting results are consistent with the peak current results, where the current increases in a continuous and linear shape until the *D* = 28 nm particles and decreases quickly after. The plot of the peak current vs. alcohol concentration shows a linear behavior of every sample with different particle sizes (Figure 7a and data from Supplementary Information Table S1).

Figure 7a shows that the peak current is directly proportional to *C_A_*, demonstrating that the approximation in Equation (5) and (6) are consistent with experiments for not too high values of the alcohol concentration. Using this fact, by a direct calculation of the slopes in previous plots, it is possible to obtain the surface energy as a function of the particle size (Figure 7a), which is consistent with the theoretical expression:

(7) $$\sigma ≅\frac{D\;RT}{4\bar{V_{p}}}ln\left(\frac{i_{p}}{i_{0}}\right)$$

The adsorption–desorption equilibrium constant (*K*_*eq*_) is obtained after the first iteration, and the results show an approximately constant value for each particle size (Figure 7b).

**Figure 8:**
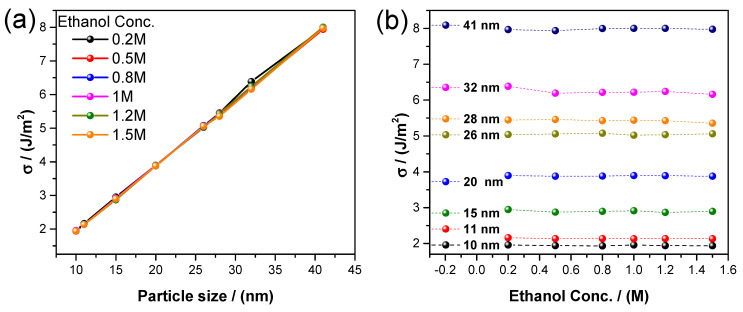
(**a**) Surface energy vs. particle size and (**b**) surface energy vs. ethanol concentration in alkaline media.

Figure 8a shows a relevant feature, that is, that the surface energy depends linearly on the particle size, as predicted by Equation (7) for all the sizes studied, and it is independent of the synthesis method, thus suggesting a universal behavior. Furthermore, the surface energy values obtained are consistent with the literature [31]. The surface energy of each particle is independent of alcohol concentration, as shown in Figure 8b.

The modification of the particle size represents a significant change in the electrocatalytic properties, and the results suggest that the use of certain particle sizes increases the electrocatalytic activity for the electro-oxidation of ethanol. This is contrary to the common belief that a small particle size is better due to the higher surface area that these materials present, because such an idea does not consider the number of active sites where the alcohol molecules should be adsorbed to promote electro-oxidation. Furthermore, the linear behavior of surface energy corroborates the dependency on particle sizes and their positive values are related to an energy that describes the classical desorption phenomenon.

## 6. Conclusions

The modification of the physicochemical properties of electrocatalytic materials, especially the particle size parameter, can improve the electrocatalytic performance. By encouraging a greater adsorption of ethanol molecules on active sites, the ethanol electro-oxidation activity grows with surface energy, as demonstrated above. This can be graphically represented as a peak current increment when the particle size reaches 28 ± 1.28 nm. For Pd/C ethanol electro-oxidation, contrary to common belief, a smaller particle size does not necessarily improve the electrocatalytic activity. On the other hand, when the particle exceeds the 28 nm optimal size found in this study, the peak current slightly drops, as can be demonstrated by the obtained results. 

It can be noticed that the theoretical fittings are good only for the first half of the oxidation curve until it reaches its maximum current. This is because only adsorption–oxidation processes occur in the growing portion of the oxidation peak, as is well explained with the modified Laviron approximation. Beyond the peak, the experimental vs. theoretical data deviate, especially for the ascorbic acid synthesis samples. This effect can be associated with surface poisoning (Pd oxidation and adsorbed CO molecules, among other species), which may be triggered by the synthesis method, the cleansing, ink elaboration, deposition, array capacitance, and other involved processes until the voltammetry is obtained. However, it is remarkable that the dependence of the surface energy on the particle size found is independent of the synthesis method.

In this work, we have shown that the modified Laviron relation given by Equation (1) and its appropriate approximation Equations (5) and (6) correctly predict the linear dependence observed for the peak current on alcohol concentration. Furthermore, our theoretical approach also predicts that the dependence of the surface energy on the particle size found is linear and independent of the alcohol concentration and synthesis method.

Thus, the obtained electrocatalytic behavior does not appear to be severely affected by either of the synthesis methods and supports the theoretical analysis performed on the assumption of “ideal” spheric nanoparticles. This could indicate that the diameter is probably the most important factor in improving the catalytic efficiency of monometallic nanoparticles.

It is important to understand that the way an ethanol molecule is adsorbed and oxidized on the surface-active site and how it can block another molecule from occupying the same site could be one of the most dominant mechanisms of the entire electro-oxidation reaction. 

Furthermore, this recent path proposed to fit the first EOR curve must be applied to different shapes and/or with other Pd-based nanoparticle systems with the Laviron-modified approximation to better understand this adsorption–blocking–desorption process.

## Figures and Tables

**Table 2:** Amount of K_2_PdCl_4_ and PVP for the modification of Pd particle size.

Polyol Synthesis				Ascorbic Acid		
Sample	K_2_PdCl_4_ (mL)	PVP (mL)	Particle Size (nm)	Sample	PVP (mg)	Particle Size (nm)
Pd IV	1	1	10	Pd 4	100	11
Pd III	2	2	26	Pd 3	92.5	15
Pd II	3	3	32	Pd 2	75	20
Pd I	4	4	41	Pd	62.5	28

## Data Availability

The original contributions presented in this study are included in the article/Supplementary Materials. Further inquiries can be directed to the corresponding author.

## Supplementary Materials

The following supporting information can be downloaded at: https://www.mdpi.com/article/10.3390/nano14231966/s1, Table S1. Experimental values used in the iteration sequences for the Laviron and modified Laviron equation. Table S2. Values of surface energy, number of adsorbed molecules and constant adsorption reaction.
