# Supplementary material for: Thermodynamic Analysis of Size-Dependent Surface Energy in Pd Nanoparticles for Enhanced Alkaline Ethanol Electro-Oxidation
Source: Nanomaterials (Basel). 2024 Dec 7;14(23):1966. doi: 10.3390/nano14231966 (PMC11643277; doi:10.3390/nano14231966)

Table S1. Experimental values used in the iteration sequences for the Laviron and modified Laviron equation.

Particle Size (nm)	Electro-chemical parameters	Concentration (mol)					
		0.01	0.025	0.04	0.05	0.06	0.075
10	$E_{1/2}$ / (V)	-0.163	-0.168	-0.167	-0.147	-0.142	-0.132
	$i_{1/2}$ / (A)	6.29x10^{-4}	7.76x10^{-4}	9.27x10^{-4}	1.44x10^{-4}	1.77x10^{-3}	2.14x10^{-3}
	E_p / (V)	-0.0392	-0.0375	-0.0338	-0.0214	-0.012	-0.008
	i_p / (A)	9.68x10^{-4}	0.00122	0.00148	0.00244	0.00308	0.0038
11	$E_{1/2}$ / (V)	-0.2	-0.173	-0.124	-0.1158	-0.0995	-0.08311
	$i_{1/2}$ / (A)	2.398x10^{-4}	8.303x10^{-4}	0.00136	0.002135	0.002808	0.0034
	E_p / (V)	0.0176	0.0284	0.04662	0.04035	0.004894	0.005634
	i_p / (A)	4.799x10^{-4}	0.001727	0.002778	0.003907	0.004894	0.005634
15	$E_{1/2}$ / (V)	-0.201	-0.1735	-0.1011	-0.086	-0.1100	-0.08774
	$i_{1/2}$ / (A)	2.034x10^{-4}	6.767x10^{-4}	0.00191	0.00259	0.002826	0.002776
	E_p / (V)	0.0601	0.02846	0.05969	0.07567	0.09514	0.1121
	i_p / (A)	8.94x10^{-4}	0.001301	0.003246	0.004224	0.000551	0.005973
20	$E_{1/2}$ / (V)	-0.201	-0.1678	-0.119	-0.0829	-0.0699	-0.0648
	$i_{1/2}$ / (A)	3.57x10^{-4}	9.587x10^{-4}	0.00173	0.00235	0.00254	0.00292
	E_p / (V)	0.0103	0.0444	0.06196	0.07018	0.07022	0.08661
	i_p / (A)	9.597x10^{-4}	0.002035	0.003185	0.003876	0.004149	0.005018
26	$E_{1/2}$ / (V)	-0.170	-0.140	-0.128	-0.116	-0.111	-0.107
	$i_{1/2}$ / (A)	6.8x10^{-4}	0.00139	0.00192	0.00233	0.00267	0.00306
	E_p / (V)	-0.042	-0.022	-0.0162	-0.0114	-0.00985	-0.00467
	i_p / (A)	0.00104	0.00239	0.00338	0.00415	0.00482	0.00558
28	$E_{1/2}$ / (V)	-0.1190	-0.1084	-0.08079	-0.06627	-0.04103	-0.02106
	$i_{1/2}$ / (A)	9.311x10^{-4}	0.001717	0.002429	0.002894	0.003348	0.003775
	E_p / (V)	0.04226	0.08367	0.143	0.17803	0.222	0.2586
	i_p / (A)	0.001975	0.00343	0.0049	0.005887	0.00706	0.008005
32	$E_{1/2}$ / (V)	-0.1858	-0.1538	-0.145	-0.1427	-0.1522	-0.13051
	$i_{1/2}$ / (A)	5.98x10^{-4}	9.16x10^{-4}	0.0012	0.00151	0.001.61	0.00222
	E_p / (V)	-0.0358	-0.0212	-0.01504	-0.01076	-0.00828	-0.00356
	i_p / (A)	0.00076	0.00135	0.00186	0.00242	0.00259	0.00372
41	$E_{1/2}$ / (V)	-0.2322	-0.2197	-0.1896	-0.1728	-0.1591	-0.14764
	$i_{1/2}$ / (A)	7.15x10^{-4}	7.6x10^{-4}	0.00129	0.00172	0.00212	0.00245
	E_p / (V)	-0.05564	-0.0381	-0.0236	-0.01781	-0.01519	-0.01068
	i_p / (A)	0.00123	0.00136	0.0024	0.00324	0.00404	0.00469

Table S2. Values of surface energy, number of adsorbed molecules and constant adsorption reaction.

Electro-chemical Results	Conc. (M)	Particle Size (nm)							
		10	11	15	20	26	28	32	41
Number of adsorbed molecules Γ_0 (mol)	0.2	1.26×10^{-8}	8.06×10^{-9}	1.12×10^{-8}	1.28×10^{-8}	1.49×10^{-8}	2.41×10^{-8}	9.43×10^{-9}	2.7×10^{-8}
	0.5	1.69×10^{-8}	2.70×10^{-8}	2.16×10^{-8}	3.27×10^{-8}	3.73×10^{-8}	5.27×10^{-8}	1.80×10^{-8}	3.21×10^{-8}
	0.8	2.14×10^{-8}	3.71×10^{-8}	4.91×10^{-8}	4.98×10^{-8}	5.28×10^{-8}	8.71×10^{-8}	2.68×10^{-8}	5.46×10^{-8}
	1	3.71×10^{-8}	5.31×10^{-8}	6.75×10^{-8}	5.78×10^{-8}	6.25×10^{-8}	1.13×10^{-7}	3.65×10^{-8}	7.04×10^{-8}
	1.2	4.99×10^{-8}	6.59×10^{-8}	8.81×10^{-8}	5.73×10^{-8}	7.2×10^{-8}	1.42×10^{-7}	4.16×10^{-8}	8.40×10^{-8}
	1.5	6.22×10^{-8}	7.67×10^{-8}	8.99×10^{-8}	7.07×10^{-8}	8.46×10^{-8}	1.71×10^{-7}	5.78×10^{-8}	9.39×10^{-7}
Adsorption reaction constant K_{eq}	0.2	1.60×10^{-7}	1.87×10^{-6}	2.60×10^{-6}	2.96×10^{-6}	1.74×10^{-7}	5.59×10^{-6}	1.10×10^{-7}	3.16×10^{-7}
	0.5	7.97×10^{-8}	2.51×10^{-6}	2×10^{-6}	3.03×10^{-6}	7.84×10^{-8}	4.89×10^{-6}	8.42×10^{-8}	1.38×10^{-7}
	0.8	6.33×10^{-8}	2.15×10^{-6}	2.85×10^{-6}	2.89×10^{-6}	1.55×10^{-7}	5.05×10^{-6}	7.80×10^{-8}	1.61×10^{-7}
	1	8.75×10^{-8}	2.46×10^{-6}	3.13×10^{-6}	2.68×10^{-6}	1.46×10^{-7}	5.26×10^{-6}	8.52×10^{-8}	1.66×10^{-7}
	1.2	9.82×10^{-8}	2.55×10^{-6}	3.41×10^{-6}	2.22×10^{-6}	1.40×10^{-7}	5.49×10^{-6}	8.09×10^{-8}	1.65×10^{-7}
	1.5	9.79×10^{-8}	2.37×10^{-6}	2.78×10^{-6}	2.19×10^{-6}	1.32×10^{-7}	5.28×10^{-6}	8.98×10^{-8}	1.49×10^{-7}
Surface Energy σ (J/m^2)	0.2	1.958	2.160	2.947	3.895	5.040	5.445	6.385	7.968
	0.5	1.939	2.133	2.872	3.877	5.055	5.457	6.192	7.940
	0.8	1.933	2.133	2.895	3.880	5.074	5.427	6.214	7.995
	1	1.956	2.132	2.911	3.894	5.020	5.435	6.217	8.000
	1.2	1.939	2.133	2.867	3.891	5.035	5.427	6.241	8.001
	1.5	1.935	2.132	2.893	3.877	5.062	5.354	6.159	7.976

The next figures show the fitting curves for the theoretical results obtained from the iteration sequences compared with the experimental results at different particle sizes.

10 nm

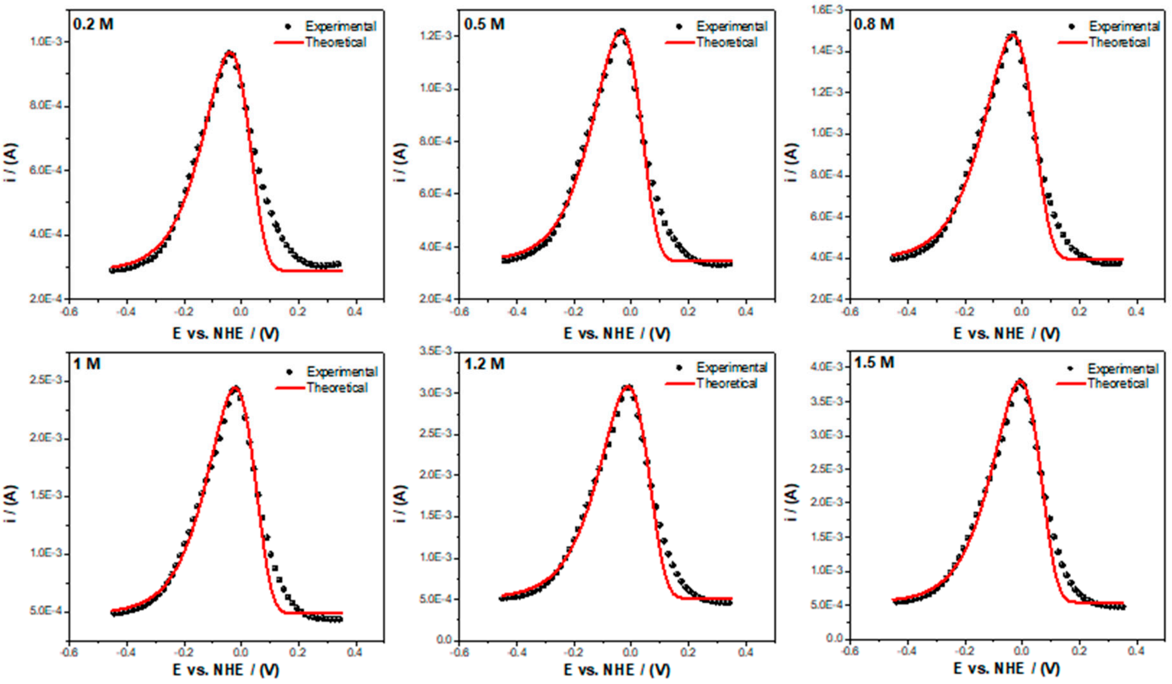

0.2 M
0.5 M
0.8 M
1 M
1.2 M
1.5 M
Experimental
Theoretical
i / (A)
E vs. NHE / (V)

11 nm

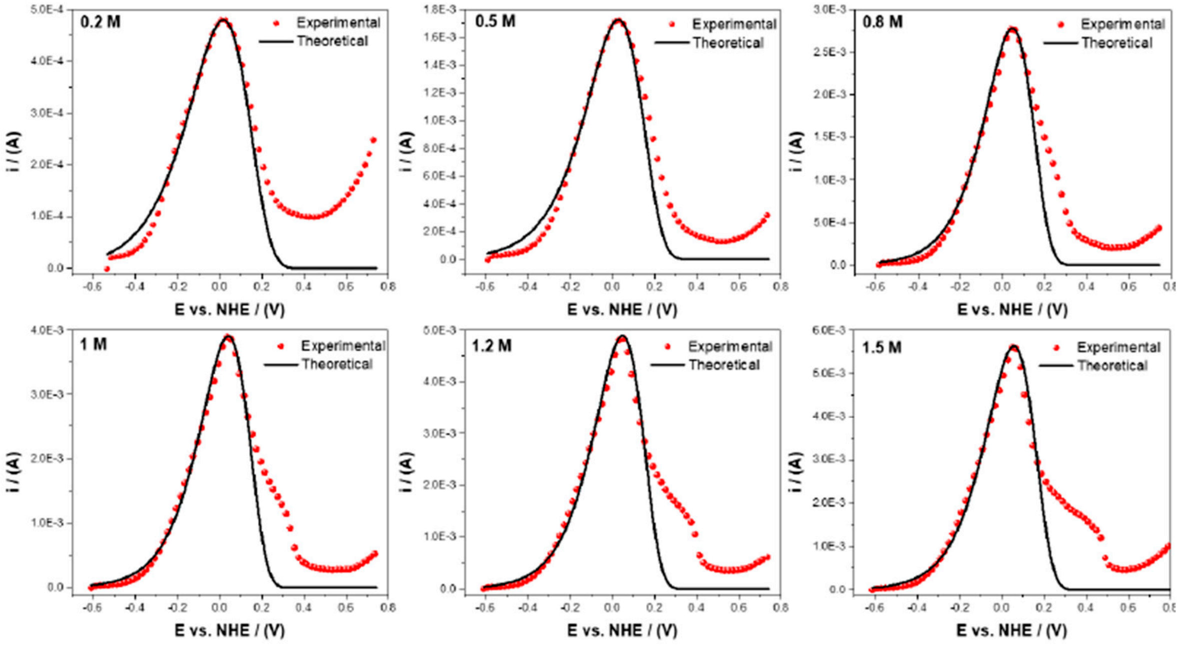

0.2 M
0.5 M
0.8 M
1 M
1.2 M
1.5 M
Experimental
Theoretical
i / (A)
E vs. NHE / (V)

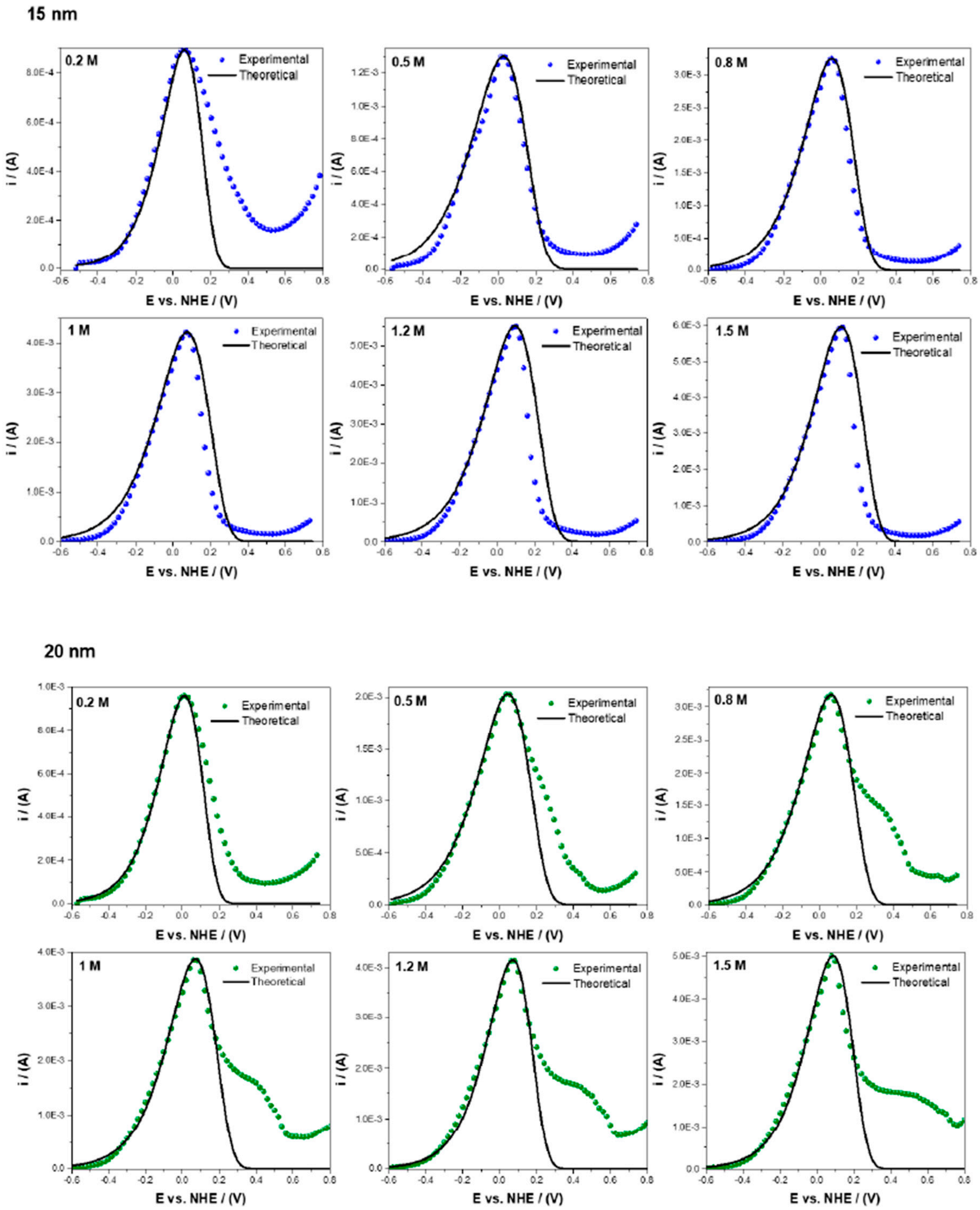
15 nm
0.2 M
0.5 M
0.8 M
1 M
1.2 M
1.5 M
Experimental
Theoretical
i / (A)
E vs. NHE / (V)
20 nm
0.2 M
0.5 M
0.8 M
1 M
1.2 M
1.5 M
Experimental
Theoretical
i / (A)
E vs. NHE / (V)

26 nm

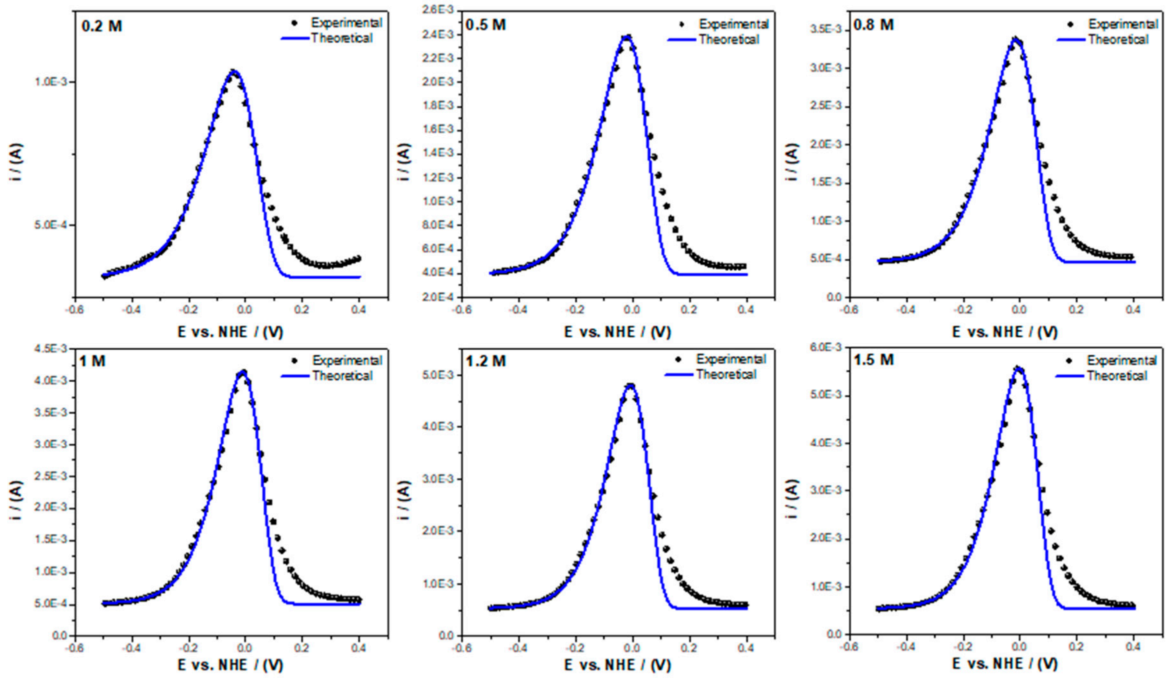
0.2 M
0.5 M
0.8 M
1 M
1.2 M
1.5 M
Experimental
Theoretical
i / (A)
E vs. NHE / (V)

32 nm

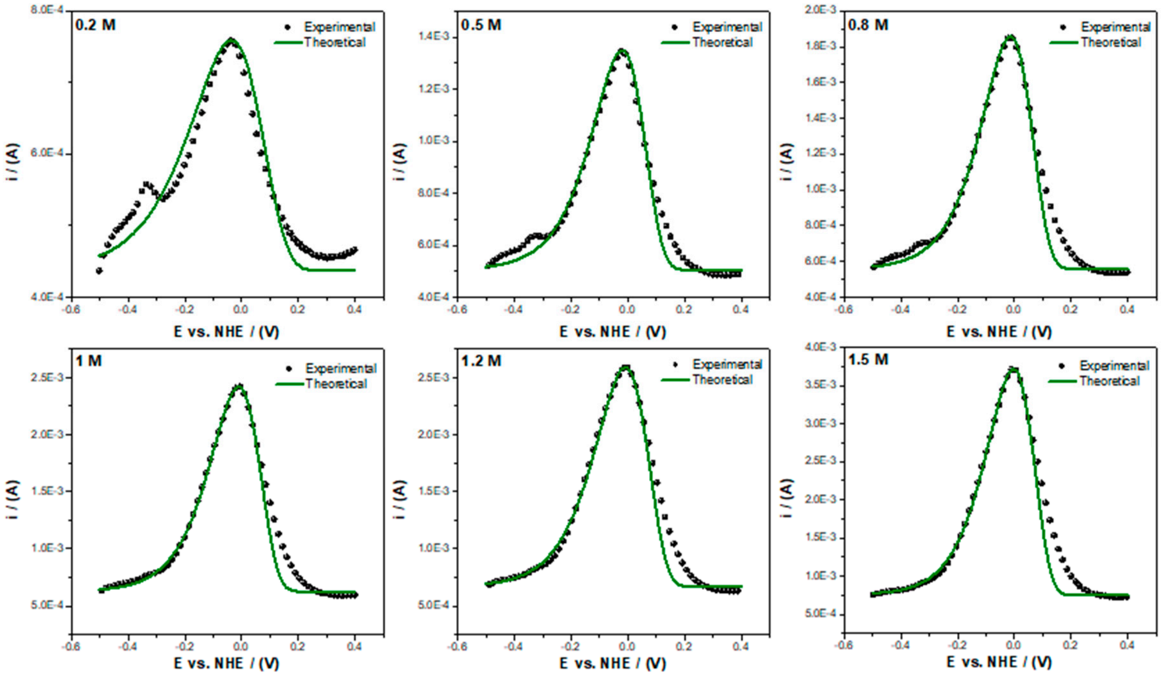
0.2 M
0.5 M
0.8 M
1 M
1.2 M
1.5 M
Experimental
Theoretical
i / (A)
E vs. NHE / (V)

41 nm

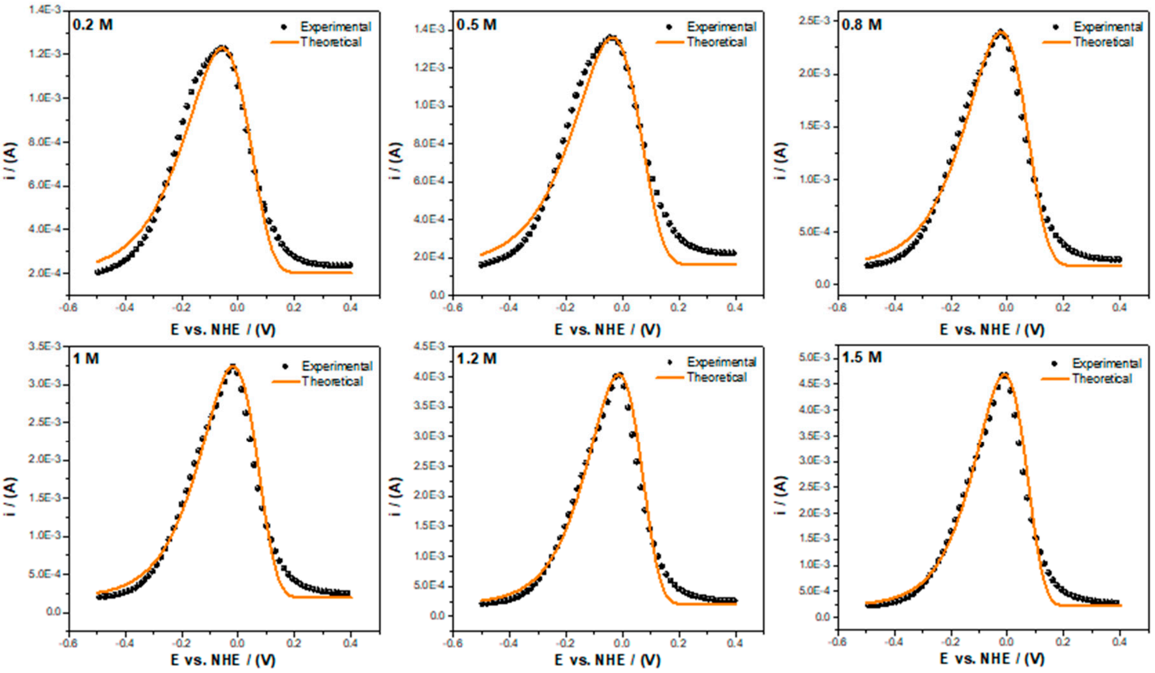

Supplement: Supplementary file 1 [file nanomaterials-14-01966-s001.zip › nanomaterials-3327469-supplementary.pdf]
